# Advertising—Our Colaborer Speaks Again

**Published:** 1876-04

**Authors:** 


*Advertising—Our Colaborer Speaks Again.*—

SHADY GROVE, April 5, 1876.

I am pleased to see that the remarks in my last upon the importance of an advertising department in a Medical Journal meets your approval, as you do me the honor to copy them.

I wish further to say that not only is it of interest and importance to medical men to see advertisement pertaining to medicine and surgery, but also to other and miscellaneous subjects. There are doubtless some of your readers, in remote sections of the country, who do not take the daily newspapers, and may not have the ready facilities for obtaining information. They would doubtless approve of a general or miscellaneous advertising department in the RECORD. We, of course, do not mean to include nostrums, or anything not likely to promote the public good. But anything useful and interesting in the line of agriculture, the arts, mechanics, manufactures, etc. There are matters of general interest, and physicians, not less than other people, like to read such things, not alone for their own benefit, but also as matters of interest and diversions which they communicate to their patrons in their daily round of practice.

I hope that the readers of the RECORD will take an interest in this matter, and aid the editors by using their influence to procure miscellaneous advertising, as well as those pertaining to medicine.

I trust also your book notices will not be neglected. Reviews of new works, and late editions of old ones, are useful to the practitioner, and add greatly to the interest of the journal.

It were well also that information in regard to medical colleges should be given. It is a matter of surprise that the various colleges do not advertise their claims. The first-class colleges should keep *standing advertisements* The brief announcements they make are frequently overlooked. The medical student and his preceptor are often in need of satisfactory and timely information in regard to the merits of the various schools. The college that sends out a notice a short while only before the opening of the sessions frequently loses students who are apt to make a selection months in advance.

You will, I trust, pardon my frequent allusion to these things as I feel interested in our journal, and desire to see it complete in all its departments. Much that is practical and useful may be done by editorial notices of this character. Remarks upon such topics and upon moral and medical ethics are important and will not fail to interest and benefit the readers of the RECORD. I hope that you will continue to give "line upon line and precept upon precept" in regard to these varied topics of interest to our medical brethren.

At an early day I propose to submit some thoughts upon "*The ways and means*" *of procuring practice.*

[NOTE.—We certainly accord fully with the views above expressed by our colaborator. We have, indeed, long entertained and urged the same views. And we shall endeavor to enlarge our advertising department by soliciting and accepting everything in the advertising line which we have reason to believe will prove useful and interesting to our readers.

In regard to medical advertisements, we would here reiterate what we have often said before, that it is of the utmost importance to the profession that the druggist and pharmaceutist who can give satisfactory assurance of their *reliability* should advertise their houses in the legitimate medical journals of the country. Physicians have learned to their sorrow that there are very many spurious extracts and preparations now in the market, so much so as to seriously weaken confidence in medicine as a science.

A writer in a pharmaceutical journal, for instance, reports the result of examination of eighteen different fluid extracts of belladonna made by different manufactories; they ranged from 410 to 80; or in other words, the weakest preparation was but one-fifth the strength of the strongest, and it is no doubt true that similar uncertainty prevails throughout the entire list of pharmaceutional preparations. This is a startling fact, and it is high time that the attention of the profession should be drawn to this matter.

The remedy for this is for the manufacturers of pharmaceutical preparations to advertise their houses through the medical journals, giving such testimonials as will satisfy medical men of the genuineness of their articles, that they may recommend them to their home druggists, who retail them to the physicians and the people generally.

If this policy was pursued it would be better for all parties. Druggists would not so often be imposed upon by venders of trash and spurious articles, and the physicians and people would reap the benefits of an improved and more successful practice.

If we had space we could give some startling facts in regard to the dangerous quality of the alcohol used by druggists generally in the preparation of tincture, bitters, etc. We would be pleased to have an article upon this subject from some one of the many readers of the RECORD.

We shall, as suggested by our colaborer, continue our efforts to enlarge and utilize our editoral department.

We shall await with interest for the appearance of his promised article on "The ways and means of procuring practice," and would be pleased to receive any items or suggestions of a practical and useful character from any of our readers. P.

